# A first approach for an evidence-based *in vitro* digestion method to adjust pancreatic enzyme replacement therapy in cystic fibrosis

**DOI:** 10.1371/journal.pone.0212459

**Published:** 2019-02-22

**Authors:** Joaquim Calvo-Lerma, Victoria Fornés-Ferrer, Irene Peinado, Ana Heredia, Carmen Ribes-Koninckx, Ana Andrés

**Affiliations:** 1 Universitat Politècnica de València, Research Institute of Food Engineering for Development, Valencia, Spain; 2 Instituto de Investigación Sanitaria La Fe de Valencia, Valencia, Spain; National Institute for Agronomic Research, FRANCE

## Abstract

**Background:**

Patients with cystic fibrosis have to take enzymatic supplements to allow for food digestion. However, an evidence-based method to adjust Pancreatic Enzyme Replacement Therapy (PERT) is inexistent, and lipid content of meals is used as a rough criterion.

**Objective:**

In this study, an *in vitro* digestion model was set up to determine the theoretical optimal dose (TOD) of enzymatic supplement for a selection of foods, which is the dose that allows for maximum lipolysis extent.

**Methods:**

A static in vitro digestion model was applied to simulate digestion of eight foods covering a wide range of lipid contents. First, the dose of the enzymatic supplement was fixed at 2000 lipase units per gram of fat (LU/g fat) using intestinal pH and bile salt concentration as variables. Second, intestinal pH and bile salt concentrations were fixed and the variable was the dose of the enzymatic supplement. Lipolysis extent was determined by measuring the free fatty acids released from initial triglycerides content of foods after digestion. Results in terms of percentage of lipolysis extent were fitted into a linear-mixed segmented model and the deducted equations were used to predict the TOD to reach 90% of lipolysis in every food. In addition, the effect of intestinal pH and bile salt concentration were investigated.

**Results:**

The predictive equations obtained for the assessed foods showed that lipolysis was not only dependent on the dose of the enzyme supplement or the lipid content. Moreover, intestinal pH and bile salt concentration had significant effects on lipolysis. Therefore an evidence-based model can be developed taking into account these variables.

**Conclusions:**

Depending on food characteristics, a specific TOD should be assigned to achieve an optimal digestion extent. This work represents a first step towards an evidence-based method for PERT dosing, which will be applied in an *in vivo* setting to validate its efficacy.

## Introduction

Exocrine pancreatic insufficiency is an associated disorder to Cystic Fibrosis (CF) affecting 85–90% of the patients [1]. The obstruction of the pancreatic duct in CF leads to a decrease in the secretion of sodium bicarbonate and pancreatic juice containing digestive enzymes to the intestine, causing nutrient maldigestion and malabsorption [2–4].

Currently, Pancreatic Enzyme Replacement Therapy (PERT) consists of the exogenous administration in every meal of a porcine-origin enzymatic supplement to promote nutrients digestion and absorption [5]. The implementation of PERT in the regular treatment of the CF led to a great improvement of nutrients digestion and absorption. However, it has been shown that patients are not able to achieve and maintain satisfactory levels of fat absorption over the years [6,7]. Clinical trials aimed at elucidating maldigestion in CF have led to inconsistent conclusions [6], and despite the fact that several authors have highlighted the need of generating knowledge to establish an accurate method, an evidence-based method for PERT dosage remains inexistent [8,9]. Therefore, lipid content of meals and/or patients’ body weight is currently the only available parameter to roughly guide health professionals and patients to adjust PERT doses. The new European Guidelines in CF council a range of 2000–4000 lipase units per gram of fat (LU/ g fat) to be supplemented in every meal that the patients take, although acknowledging no evidence for this recommendation [10].

Moreover, a recent study has elucidated that there are huge differences in the dosing of PERT among European countries, showing large variability within and between patients, without association with weight or body mass index [11]. This pattern of intake compromises the recommendations of adjusting the enzymes dose to the lipid content of the meals, since this lipid content largely varies from one meal to another and from one day to another [11].

Furthermore, intrinsic food factors, have been pointed out as determinants in the process of gastrointestinal nutrient digestion and thus in the effectiveness of the enzyme supplements[5,8,12–16]. Food matrix physicochemical properties or lipids origin have proved to highly influence the process of lipids hydrolysis [16–18]. Likewise, the gastrointestinal environmental conditions have demonstrated to play a key role on lipids digestion [8,16,19–21]. In the particular case of CF, specific differences as compared to the healthy population take place in the intestine (pH and bile salt concentration) due to the pancreatic insufficiency and the altered biliary function [18,19,22].

In this context, *in vitro* digestion studies of different foods might be a useful tool to shed light on the understanding of lipolysis in CF and to give guidance on enzymatic supplement dosing. They allow for the controlled, accurate and reproducible in-lab simulation of the physiological gastrointestinal conditions [23]. Nevertheless, up to now, there were only a few known investigations about lipid digestion in real or complex foods, which limits the translation of knowledge from *in vitro* digestion outcomes to clinical practice [16,24,25].

In the above-described scenario, MyCyFAPP Project (www.mycyfapp.eu) pursues CF patients’ self-management of PERT supported by a mobile application by means of a new dosing predictive model [26]. Within the frame of MyCyFAPP, the aim of this study is setting up an evidence-based method for PERT adjustment by applying a static *in vitro* digestion method to obtain the theoretical optimal dose of enzymatic supplements (TOD) for a selection of foods.

## Material & methods

### Materials and test foods

Pancreatic enzyme supplements (Kreon 10,000 LU), were kindly donated by “Hospital Universitari Politècnic La Fe” (Valencia, Spain). Each capsule contains 150 mg of porcine pancreatic enzymes in the shape of gastro-resistant microspheres equivalent to 10,000 lipase U, 8,000 amylase U, and 600 protease U. One lipase unit is defined as is defined as the quantity of enzyme that will liberate 1 μmol of butyric acid per minute under the conditions of the test (tributyrin as substrate, pH 7.0 and 30°C) [27]. All the capsules used in the study belonged to the same batch. Routine enzymatic activity assays were carried out daily to follow up lipase activity [27]. The other chemicals used for the *in vitro* digestion were: pepsin from porcine gastric mucosa (3200–4500 U/mg), bovine bile extract (Sygma Aldrich, CAS number: 8008-63-7), KCl, KH_2_PO_4_, NaHCO_3_, NaCl, MgCl_2_ (H2O)_6_, (NH_4_)_2_CO_3_ y CaCl_2_ and Triton X-100 obtained from Sigma-Aldrich Chemical Company (St Louis, MO, USA), Ethanol obtained from Vidrafoc (Barcelona, Spain) and NaOH, 1 N and HCl 1 N acquired from *AppliChem Panreac*. Food products were purchased at a local supermarket.

A total of eight foods covering a wide range of lipid contents were selected: salad with olive oil (9.4% lipids), pizza (7.4%), Greek-style yoghurt (10%), ham and cheese sandwich (7.9%), milk (3.6%), buttery bread (24%), breakfast cereal (4%) and chocolate biscuits (27%). The selection was made upon some of the most consumed food products identified in a paediatric CF population (N-P-037, ESPGHAN 50^th^ Annual Meeting). It included different food structures (i.e. emulsions, fibres, proteinaceous, etc.), with different lipid types (animal and vegetal origin).

### Study design

Two sets of experiments were conducted for each of the tested food products. The first one was aimed at elucidating the influence of the gastrointestinal factors on the lipolysis extent. The selection of the intestinal pH and bile salt concentration to study was based on a thorough literature research. The average intestinal pH in CF can be considered 6, while 7 can be representative of healthy populations [18,23,28,29]. In the case of bile salt concentration, CF patients have shown to have up to 10 times lowered the concentration found in healthy individuals [23,30,31]. Therefore, in this set, the dose of the enzymatic supplement was fixed at 2000 LU/g fat and intestinal pH and bile salt concentration were the variables with two levels each: pH 6 and pH 7, and bile salt concentration 1 mM and 10 mM, giving four pH/bile combinations: 6/1, 6/10, 7/1 and 7/10.

The second set of experiments was established to assess the effect of the dose of the enzymatic supplement under fixed gastrointestinal conditions. These conditions were selected as the worst possible scenario in the small intestine digestion in CF patients, namely: the altered biliary function leads to a lower bile salt concentration in the intestine [30,32], and the obstruction of the pancreatic duct to a lower pH as a consequence of the lower secretion of sodium bicarbonate [2,3,22,33,34]. So, intestinal pH and bile salt concentrations were fixed at 6 and 1mM respectively and the variable was the dose of the enzymatic supplement, with five levels: 0, 1000, 2000, 3000 and 4000 LU/ g fat. Results from this experimental set were modelled to obtain predictive equations for TOD calculation by means of regression models.

All the experimental conditions and analyses were conducted at least in triplicate.

### In vitro digestion simulation

The digestion process was simulated according to the static standardised method proposed by Minekus et al., (2014) [23] which establishes the “smallest common denominator” of the standard conditions that are close to the physiology of a healthy adult, and thereafter amendments were applied according to the scope of this research ^23^. The static digestion process was simulated in three stages.

#### Oral stage

The food samples (5 g), were mixed in a proportion 1:1 (w/v) with simulated salivary fluid (SSF), and properly homogenized during 2 minutes. The mixture was then placed in 50 mL falcon tubes and incubated for 5 min at 37°C.

#### Gastric stage

Then, simulated gastric fluid (SGF) (pH 3) was added in a proportion 1:1 (w/v) to each tube containing the oral bolus. The pH of the mixtures was readjusted to pH 3 with HCl (1N). Pepsin was added into the SGF to reach a concentration in the gastric mixture of (2000 U/mL). At this point, PERT was added into the tubes in a concentration established in the experimental design. Gastric lipase was not used in the experiment as recommended by the Infogest International Protocol for in vitro digestions [23]. The reasons supporting this decision include the scarce commercial availability of the product and its low activity (optimal at pH 5 and easily inactivated by gastric pepsin). Samples were rotated head-over-heels at 55 rpm for 2 h at 37°C using an Intell-Mixer RM-2 (Elmi Ltd, Riga, LV-1006, Latvia) and an incubator chamber Selecta (JP Selecta SA, Barcelona). These mixing conditions provided constant mechanical energy to induce the breakdown of the food matrixes during digestion, as occurs in the physiological process.

#### Intestinal stage

Following the gastric stage, simulated intestinal fluid (SIF) (pH 6), was added in a proportion 1:1 (w/v) to each tube containing the gastric chyme. Bile was added to the SIF in order to reach a final concentration in the intestinal mix of either 1 mM or 10 mM depending on the experimental design. The pH of the mixtures was adjusted, with NaOH (1N), to pH 6 or 7, depending on the experimental design. The samples were then rotated head-over-heels at 55 rpm for other 2 h at 37°C. pH was monitored during the digestion process and readjusted if necessary as pH below 5.7 might inactivate lipase activity [23,24]. After two hours of digestion, samples were placed in an ice bath for ten minutes and pH was adjusted to 9 to stop enzymatic activity [35,36].

Fluids’ composition required for each digestion stage, were those described by Minekus et al., 2014 [23]; they were prepared fresh daily and kept at 37°C before their use.

### Lipolysis analysis

Lipolysis extent was determined by measuring the free fatty acids (FFA) released from initial triglycerides content of foods after the complete simulated gastro intestinal digestion. The liquid phase from the digested samples (100 μl) was separated by sieving (1 mm^2^) and mixed with 10 mL of a solution made of 5.6% Triton X-100 and 6% ethanol in water, to solubilize the free fatty acids and ensure to stop lipase activity [37]. In addition digestion tubes were put in ice immediately after the intestinal stage simulation to guarantee the rapid inactivation of enzymatic activity. In preliminary experiments we checked the activity was stopped, by means of assessing lipolysis after 30 minutes from the end of the intestinal stage, with no differences observed. The free fatty acids were measured on the diluted samples using a free fatty acid spectrophotometric assay kit (Roche Diagnostics, Indianapolis, IN, USA) in a spectrophotometer (UV/vis, *Beckman Coulter*) [37]. Palmitic acid standard was used for quantitative determination of FFA. Digested fat was estimated assuming the release of 2 moles of fatty acids and 1 mole of moloacylglyceride per 1 mole of triglycerides [37]. Values higher than 100% may be related to the fact that the TAG hydrolysis level can be higher than 66.6% (which correspond to the release of 2 FFA) as some remaining monoacylglycerides can be converted into glycerol.

### Statistical analyses

Experimental data were summarised by mean (standard deviation) in case of continuous variables (dose of enzymatic supplement) and by relative and absolute frequencies in case of categorical variables (pH, salt concentration). The association studies between lipolysis and the intestinal conditions (pH and bile salt concentration) at the standard dose of 2000 LU/g fat were analysed by linear mixed regression considering each different food as a random factor. These models were fitted for the logarithmic-transformed lipolysis extent to normalize the response and guarantee positive fitted values with the regression equation. The results of the model can be interpreted with the estimated effect (i.e., estimate) and the 95% confidence interval (95% CI). If the estimate is > 0 the variable is positively associated with the response variable, i.e. lipolysis extent, and if < 1 the effect is diminishing of the response variable. The higher the value is, the higher the effect is. Complementarily, the 95% confidence intervals that do not contain 0 are those significantly associated with the response variable.

The estimation of the dose of enzymatic supplement that maximises lipolysis extent (i.e., the TOD) and the predictive equations of these models were solved numerically using segmented mixed models with random change points for each food [38].

The difference in the dose of enzymatic supplement between the application of the predictive linear mixed regression model and the regular recommendation of the current guidelines [10] was provided by multiplying the amount of fat of a regular portion size of the test foods by the dose of the enzymatic supplement in LU.

All the analyses were performed using R software (version 3.3.3), and lme4 (version 1.1–12), nlme (version 3.1–131), and clickR (version 0.3.35) packages. A p-value below 0.05 was considered statistically significant.

## Results

### Influence of some food extrinsic factors on lipolysis extent

The mean values of lipolysis extent at pH 6 and 7 were 60.8% and 104.74%, respectively. When considering the bile salt concentration, experiments performed with 1 mM led to a mean lipolysis extent of 60.37%, while 99.96% was reached when bile formulation was 10 mM. Both variables, intestinal pH and bile salt concentration, had a statistically significant (p<0.001) influence on lipolysis. Both increasing bile salt concentration and intestinal pH had a positive effect on lipolysis (CI 95% [1.28, 1.55] and CI 95% [1.15, 1.39], respectively). In addition, a significant interaction between both factors was found (p = 0.03, IC95% [-0.28, -0.02]), meaning that food samples digested *in vitro* at intestinal pH 7 and bile salt concentration of 10 mM had not a lipolysis extent as high as expected with the combination of both effects (CI 95% [-0.28, -0.01]), (Table 1).

**Table 1:** Linear mixed regression model for food extrinsic factors: Influence of intestinal pH and bile salt concentration on lipolysis extent.

Variable		Lipolysis extent (%) Mean (SD ^a^)	Estimate [95 CI^b^ %]	P-value
Intestinal pH	pH 6	60.8 (34.19)	0.35 [0.25, 0.44]	<0.001
	pH 7	104.67 (17.81)		
Bile salt concentration	1 mM^c^	62.37 (35.41)	0.24 [0.14, 0.33]	<0.001
	10 mM	99.96 (21.27)		
Interaction pH7: 10 mM		-	-0.15 [-0.28, -0.02]	0.033

^a^SD, standard deviation

^b^CI, confidence interval

^c^mM, millimolar

These results evidence that the gastrointestinal conditions of the CF patients do affect the enzymatic supplement activity and thus the lipolysis extent.

### Influence of enzyme dose on lipolysis extent

**Fig 1:**
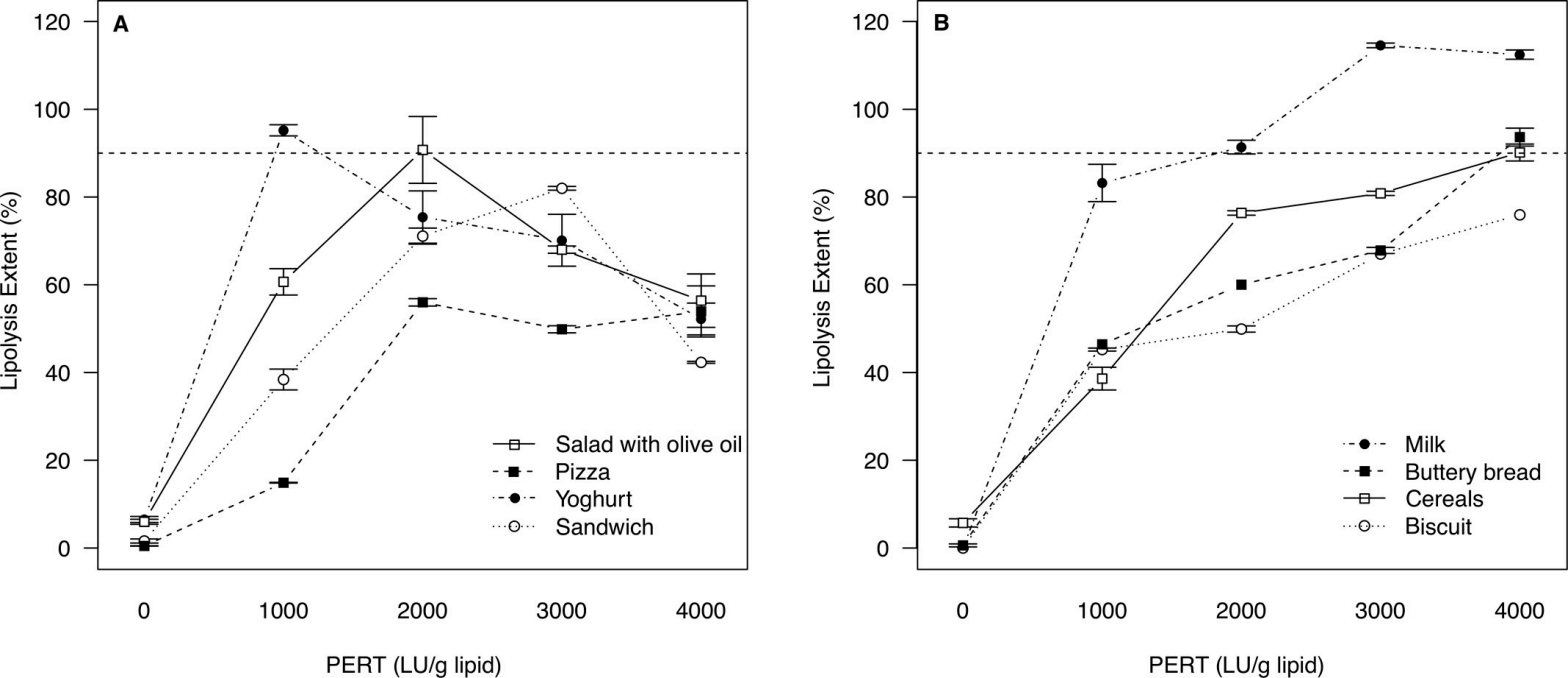
Lipolysis extent (%) versus different doses of enzymatic supplement (LU/g lipid) after *in vitro* digestion (intestinal pH 6 and 1 mM bile salt concentration). The horizontal dotted line represents a 90% of lipolysis extent target. Fig 1A: foods with a decreasing lipolysis extent with dose; Fig 1B: foods with an increasing lipolysis extent with dose.

In the selected foods, lipolysis was assessed with different doses of enzymatic supplement (0, 1000, 2000, 3000 and 4000 LU/g fat) under the most unfavourable CF intestinal conditions (pH 6 and Bile 1 mM) based on the literature [18,22,28,30,31]. According to the results of lipolysis extent as a function of the dose of the enzymatic supplement, two groups could be differentiated. One food group, consisting on salad with olive oil, pizza, yoghurt and sandwich, reached the maximum lipolysis extent at 2000 LU/g fat, with decreasing lipolysis after further increasing the dose of enzymes (Fig 1A). On the contrary, the other food group, consisting on milk, bread with butter, cereals and biscuit, exhibited an increasing lipolysis extent when increasing the dose of enzymes above 2000 LU/g fat (Fig 1B).

### Modelling lipolysis extent under the most unfavourable Cystic Fibrosis conditions

**Fig 2:**
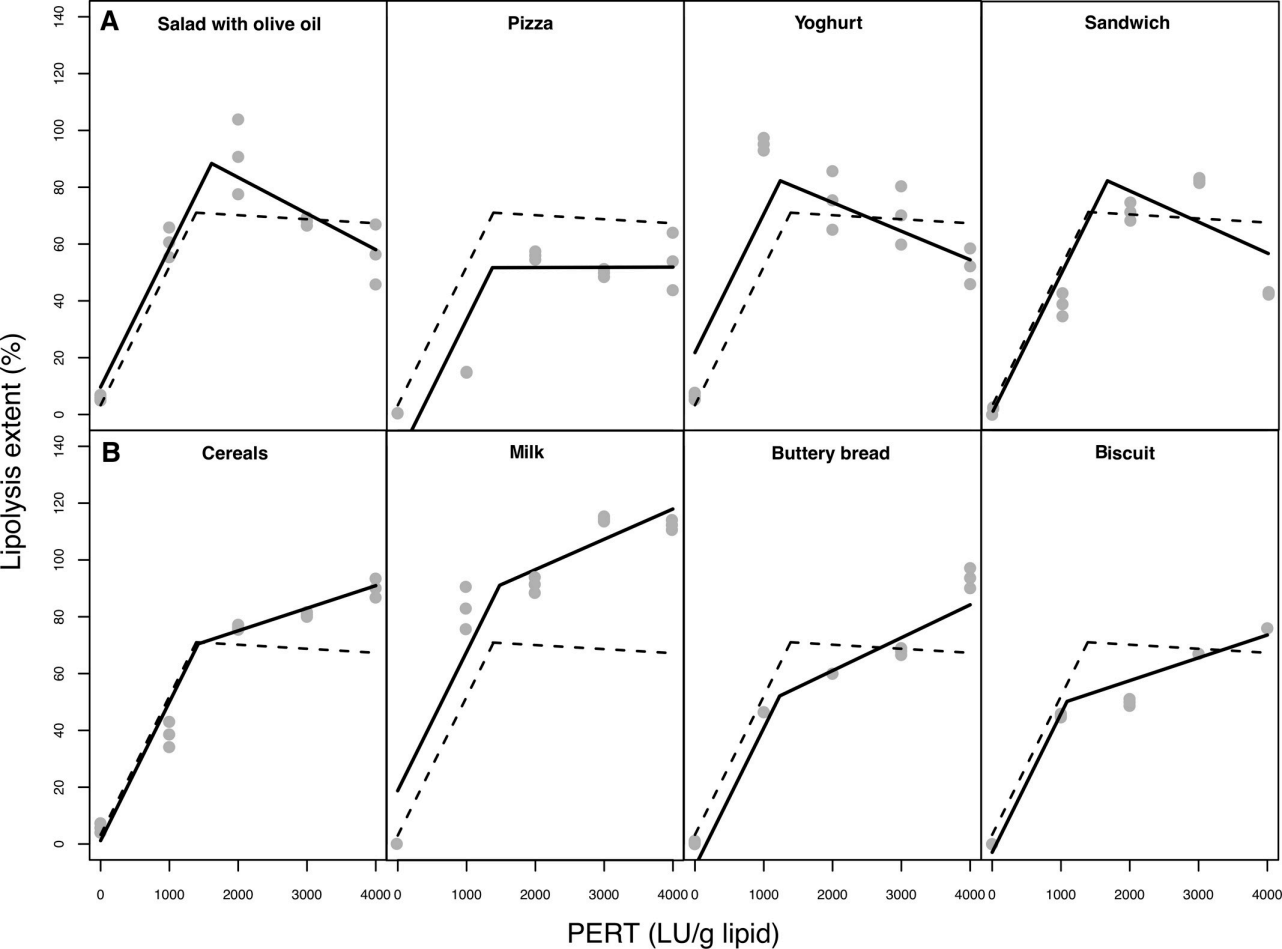
Lipolysis extent (%) as a function of PERT dose (LU/g lipid) predicted for each food by applying the segmented mixed regression model. The dotted line represents the evolution considering all the foods together and the solid line shows the estimation for each food individually. Fig 2A: foods with a decreasing lipolysis extent with dose; Fig 2B: foods with an increasing lipolysis extent with dose.

A segmented mixed regression model was applied to study and describe the association of lipolysis extent with the dose of the enzymatic supplement for each food product. By applying this model, it was possible to obtain both, the dose at which the improvement of lipolysis extension changes with dose increase (change points in the lipolysis slopes), and the different slopes after this change point for each food product. It was confirmed that each meal or food product had a change point in the dose of enzyme supplement from which the slope changes, either because it decreased, i.e. negative slope, (Fig 2A) or slightly increased, i.e. positive slope (Fig 2B). Concretely, cereal, milk, buttery bread, and biscuit showed improved lipolysis extent with the dose, while in salad with olive oil, ham and cheese pizza, yoghurt and ham and cheese sandwich the opposite tendency was found.

The data modelling provided the equations (**Eq 1**) that enable for the prediction of the optimal dose, allowing for maximum lipolysis, for each food:

$$Y=\beta_{0i}+\beta_{1}*X+\delta_{i}*(X-\varphi_{i})+\varepsilon_{i}$$ (1)

where,

“*Y*” is the lipolysis extent (%);

“*X*” is a continuous variable representing the dose of the enzymatic supplement (LU/g of lipid);

“i” refers to the type of food (i = 1 (salad), i = 2 (pizza),…, i = 8 (chocolate biscuit)).

Therefore, the equation allows for predicting the dose of the enzymatic supplement (X) for each specific food when a target lipolysis extent (Y) equal to 90% is given. Of note, the parameter X appears twice in the equation as the segmented model describes the dose-response behaviour in two parts: the first part (*β*_0*i*_ + *β*_1_ * *X*) represents the increasing slope before the change point, and the second part (+*δ*_*i*_ * (*X* − *φ*_*i*_) + *ε*_*i*_) describes the slope tendency after the change point. The rest of the parameters of the equation are explained below.

For each food *i*,

"*β*_0_” is the parameter expressing the mean response value at *X* = 0

"*β*_1_" is the slope before the change point. Since there are a very limited number of experimental data in the range between 0 and 1000 LU/g lipid, in which probably different slopes could have been obtained, the same value in all foods assessed is assumed, resulting in a slope of 0.05.

"*φ*_*i*_" is the dose of the enzymatic supplement at the change point or breakpoint which determines the change in the lipolysis extent trajectory in each food *i*.

$"\varepsilon_{i}"∼N(0,\sigma_{\varepsilon }^{2}),$ represents the non-explained variance by the regression model.

Thus, to interpret the equation, the “I” function has to be considered, which considers the dose (X) and the change point (*φ*_*i*_), i.e. (*X* − *φ*_*i*_). It is a logic expression that can equal to “0” (false) when the dose (X) is lower than the change point, or to “1” when the dose (X) after the change point is higher. Therefore, if I = 0, the second part of the equation does not apply, since “*δ*_*i*_ * (*X* − *φ*_*i*_)" would equal to 0.

Thereby, if *β*_1_ + *δ*_*i*_ > 0, the slope after breakpoint would be positive and the target 90% lipolysis extent could be reached. However, if *β*_1_ + *δ*_*i*_ < 0 the sign of the slope after change point would be negative, then the lipolysis extent could not be increased after it, and the maximum lipolysis would have been already reached in the change point.

In four out of the eight test foods, a lipolysis extent of 90 could be reached when applying the equations, and TODs could be calculated (**Table 2**). These foods were milk, buttery bread, cereal, and biscuit, and the recommended TODs ranged from 1480 to 6130 LU/g fat. In the case of milk, the TOD coincided with the dose at the change point (φi) as lipolysis extent was 90.49%. In the other three foods, lipolysis extents at the change point ranged from 50.03 to 69.22%, so their TODs increased in a direct proportion with the slope after the change point.

**Table 2:** Summary of lipolysis extent at the change point and theoretical optimal dose (TOD) to obtain lipolysis extent of 90%.

Food	Lipolysis extent (%) at change point (*φ*_*i*_)	Slope sign (*δ*_*i*_) after change point	TOD (LU/g fat) for 90% lipolysis extent
Salad with olive oil	89.95% (1613)	negative (-0.013)	-
Ham & cheese Pizza	53.73% (1375)	negative (-0.001)	-
Yoghurt	85.18% (1240)	negative (-0.011)	-
Ham & cheese Sandwich	81.04% (1660)	negative (-0.009)	-
Milk	90.49% (1480)	positive (0.011)	1480
Buttery bread	60.13% (1230)	positive (0.014)	3400
Cereal	69.22% (1420)	positive (0.006)	4720
Biscuit	50.03% (1090)	positive (0.008)	6130

Foods presenting decreasing lipolysis with dose of enzymes after the change point reached lipolysis extents higher than 80%, in the case of ham and cheese sandwich, yoghurt, and salad with olive oil. However, pizza presented the lowest lipolysis extent, which was 53.73%. These results show that some foods can theoretically reach the optimal lipolysis extent (90%) when increasing the dose of enzymes and others would not achieve higher lipolysis extents than those reached at the change point, despite an increase in the dose.

### Comparison of the predicted doses to the current recommendation

Finally, Table 3 provides an example of the TODs for the components of the test meals predicted by the linear mixed regression model as compared to the standard doses of the enzymatic supplement based on current guidelines recommendations.

**Table 3:** Theoretical optimal dose of enzymes (TOD) as compared to the recommended dose by the Guidelines for Nutrition in Cystic Fibrosis (Turck et al. 2016) [10] expressed ad LU/g fat, total LU dose and amount of 10.000 LU capsules.

	Buttery bread	Salad with olive oil	Ham & cheese sandwich	Milk	Cereal	Yoghurt	Ham & cheese pizza	Biscuit
Regular portion size (g)	30	50	100	250	40	125	200	22
Amount of fat (g)^a^	8.5	5	10.2	7.2	1.2	12.5	16.5	5.7
TOD (LU/g fat)	3400	1613	1660	1480	4720	1240	1375	6130
Standard PERT dose (LU/g fat) ^b^	2000–4000							
Total TOD dose (LU)	28900	8065	16932	10656	5664	15500	22688	34941
Standard total dose of enzymatic supplement (LU)^b^	16400–32800	10000–20000	20400–40800	14400–28000	2400–4800	25000–50000	33000–66000	11400–22800
Number of capsules of enzymatic supplement (10.000 LU) based on TOD	3	1	2	1	1	2	3	4
Number of capsules of enzymatic supplement (10.000 LU based on standard recommendation)^c^	2–3	1–2	2–4	1–3	1	3–5	3–7	1–2

^a^ Amount of fat considering a regular portion size.

^b^ Recommended dose of enzymatic supplement according to the Guidelines for Nutrition in Cystic Fibrosis (Turck et al. 2016) [10].

^c^ Approximation made by considering enzyme supplements of 10000 LU.

Considering the recommended range by the guidelines of 2000 to 4000 LU/g fat [10], the number of 10000 LU capsules for the tested foods in their regular portion sizes ranged from 1 to 7 capsules, while considering the TODs, the recommended number of capsules was 1 to 4. For buttery bread, salad with olive oil, ham and cheese sandwich, milk, cereal, and ham and cheese pizza, the predicted number of capsules calculated with the TOD was within the range of the recommended number of capsules calculated with the recommendation of the guidelines. However, the number of capsules according to TOD for these foods corresponded to the lower range (except for buttery bread), so the guidelines recommendations could be exceeding the optimal dose. On the other hand, for yogurt, the indicated number of capsules calculated by TOD, was lower than the one calculated by the standard recommendation, and it was higher for biscuit.

## Discussion

The approach presented in this study allowed establishing a scientific-based method to analyse the influence of some food intrinsic factors and some physiological conditions on lipolysis, and to predict the theoretical optimal dose of enzyme supplements (TOD) for 8 specific foods in CF specific conditions. The segmented mixed regression models were used to obtain the equations adjusted to the experimental results, which allowed for calculating an optimal enzyme dose to reach a specific lipolysis extent target. The model considered the inherent-to-food properties and the worst gastrointestinal conditions in CF patients (intestinal pH6 and bile 1 mM). In addition, complementary experiments were performed in order to evaluate the influence of other intestinal conditions on lipolysis extent.

The results showed that the intestinal pH, along with the bile salt concentration, were the major determinant factors affecting the process of lipolysis. It is well known that pancreatic lipase activity is higher at pH 7 than at pH 6 [36] and that bile salt contribute to the emulsification process of lipids in the digestive fluids and therefore increasing the interfacial surface of the lipids available for being hydrolysed [39]. This evidence suggests that proton pump inhibitors can be important as additional therapy to PERT since they would help to increase intestinal pH.

The influence of the inherent-to-food properties was evidenced when assessing the lipolysis extent against enzymes dose, at fixed gastrointestinal conditions i.e. pH 6 and bile 1 mM, and “food” was considered as a random effect. The segmented regression model showed that some of the assessed foods followed a constantly increasing relationship between enzyme dose and lipolysis extent whilst others tended to decrease after a certain dose.

Natural emulsions like milk and yoghurt, with a large interfacial surface (smaller and larger number of available fat molecules) than other solid foods, provide an easier accessibility for the enzymes to the fat globules surface [39,40]. In addition, these matrices are easily disintegrated, favouring the fat molecules release from the food matrix [41]. Therefore, these two foods were able to reach the highest extents of lipolysis under the simulated conditions. Similarly, the only lipids source in the salad was the added olive oil, this not being trapped into the matrix and thus being very accessible to enzymes (free fat). The same pattern was observed in the buttered bread, where all the lipids came mainly from the butter spread on the bread. Mechanic agitation both in gastric and intestinal stages led to fat phase separation, resulting in a digestion media where lipids are easily emulsified and accessible to the enzymes and not trapped in the food matrix.

In contrast, we observed that those foods with higher contents of complex carbohydrates were those showing the smallest slopes with increasing the dose of the enzymatic supplement: sandwich (bread) and pizza (dough). These results may be due to the carbohydrates increased the viscosity in the digestion media [13,42] and affected the enzyme-substrate adsorption process. Several studies assessing nutrient digestion in model systems have reported that the initial structure and composition of the food matrix influences the disintegration mechanism and subsequently the kinetics of nutrients release [43]. For instance, one study assessing proteolysis in yogurt found that the more viscous texture of the matrix could affect food mixing and interactions with enzymes, and contribute to the slightly lower protein digestibility during *in vitro* digestion [37]. More concretely, this phenomenon was also reported in some types of starch [44–46].

The predictive model described two possible patterns in lipolysis extent in relation to the doses of enzymatic supplement. Reaching the target value, was not dependent exclusively on the lipid content of the meals. Thus, milk (3.6% lipid) and yoghurt (10% lipid) reached > 90% lipolysis when using doses of 1000 LU/g lipid, followed by salad (9.4% lipid) that needed 2000 LU/g lipid. Noteworthy, those foods with the highest percentage of lipids, buttered bread (24%) and biscuits (27%), resulted in the highest requirements of enzymatic supplement per gram of lipid to reach the threshold of 90% lipolysis. In addition, when comparing the predicted TODs to the current guidelines recommendation (2000–4000 LU/g fat), most of the foods required TOD values between 1000 and 2000 LU/g fat, falling into the recommendation lower limit, although some of them were above upper limit recommendation. Thus, the results suggest that the range to achieve optimal lipolysis could be extended to around 1000 to 6000 LU/g of fat.

One limitation of this study is that some inherent-to-food factors (such as nutrient composition or lipid structure in the matrix) have not been taken into account as specific co-variables in the model. The reason for this was the sample size, i.e. eight foods, did not allow for robust analysis when adding variables other than the doses of enzymes. However, these characteristics have been controlled and considered in the model by means of the random effect of each food. This effect allows for controlling the non-independence of the data, which means that, for example, each food has its specific change point. Secondly, other agents than the intestinal pH, the bile salts concentration or the composition of the digestion fluids have not been studied. However, the scope of the present research relates to the lipid hydrolysis in the small intestine, which is hypothesised to be the major determinant of the efficacy of the enzymatic supplement. Other individual factors affecting lipid absorption will be considered in a subsequent study with patients as part of the present research.

The main strength of this study is, indeed, the inclusion of eight different complex foods as samples for *in vitro* digestion to study lipolysis. These foods presented different structural and compositional characteristics, and were tested under different gastrointestinal conditions. Therefore, we consider this integral approach used in the study as appropriate to generate knowledge on lipolysis extent in a multi-variable framework.

Another strength and novelty of this study is the applied statistical model. The mixed segmented model is the most adequate to describe the relationship between enzyme dose and the lipolysis extent, since two linear tendencies, separated by a change point, coexist in the same food product. The model allows for the identification of those foods for which increasing of the dose of the enzymatic supplement would lead to a higher lipolysis extent. Besides, the model can be applied to results of other foods undertaking the same experimental design, and it could be assumed that all of them will show one of the two tendencies previously described.

Therefore, the results reported in this paper may have a great potential application in clinical practice in the near future, since they will be applied in an *in vivo* clinical pilot study in paediatric patients with Cystic Fibrosis from six European CF-centres. Participants will follow a 24-hour menu consisting of the foods we studied, along with the TODs obtained in this part of the study. Collections of faecal samples will be used to calculate the coefficient of fat absorption (CFA), which is the clinical correlate of the lipolysis extent. In this way, the prediction model of TODs will be tested *in vivo*.

In conclusion, a first approach towards an evidenced-based method for PERT adjustment has been conducted. This work proves that different intrinsic-to-food factors as well as factors related to the disease-specific gastrointestinal environment will have an effect on the lipolysis extent and consecutively on the TOD. According to our results, the lipid amount in food alone is not an appropriate criterion to adjust the dose of the enzymatic supplement. Besides, each food should be assigned with a specific TOD in order to adjust enzyme supplement dosing. These findings warrant further research by the MyCyFAPP working group.

## Supporting information

S1 Table Study database. The table includes all the study variables and the response variable being lipolysis %.
(CSV)

S1 File Code for the linear mixed regression model. This model was applied to study the effect of extrinsic factors (bile salt concentration and intestinal pH) on lipolysis extent.
(R) Click here for additional data file.

S2 File Code for the segmented mixed regression model. This model was applied to study the effect of the enzyme supplement dose and to predict the theoretical optimal dose (TOD).
(R) Click here for additional data file.

S3 File Code for data treatment and statistical analyses. This file compiles all the code and commands to be executed with R software.
(DOCX)
