# Supplementary material for: A first approach for an evidence-based in vitro digestion method to adjust pancreatic enzyme replacement therapy in cystic fibrosis
Source: PLoS One. 2019 Feb 22;14(2):e0212459. doi: 10.1371/journal.pone.0212459 (PMC6386532; doi:10.1371/journal.pone.0212459)
Supplement: S3 File — This file compiles all the code and commands to be executed with R software. (DOCX) [file pone.0212459.s004.docx]

######### Load libraries ############

library(clickR)

library(lme4)

library(nlme)

library(sciplot)

#run file source-segmented.lme.r

######### Influence of some extrinsic factors on lipolysis extent

datos<-read.csv2('data_base.csv')

datos<-nice_names(datos)

names(datos)

datos[,c(3,10,11)]<-fix.factors(datos[,c(3,10,11)])

report(datos[,c('lipolysis','bile.salt.concentration')],by='bile.salt.concentration')

report(datos[,c('lipolysis','ph_intst')],by='ph_intst')

datos$log_lipolysis<-log(datos$lipolysis)

fit_model<-lmer(log_lipolysis~bile.salt.concentration*ph_intst+(1|Food), data=datos[datos$lu_g_fat==2000,])

report(fit_model,file='report_fit_model')

report(fit_model)

exp(cbind(c(4.245,0.238,0.346,-0.148),c(4.139,0.144,0.252,-0.28),c(4.352,0.332,0.44,-0.015)))

######### Modelling lipolysis extent under Cystic Fibrosis conditions

datos2<-read.csv2('datos_segmented090318.csv')

names(datos2)

datos2$FOOD<-factor(datos2$FOOD,levels=c('Salad','Milk','Pizza',' Bread & butter','Greek Yogurt','Breakfast cereal','Ham & Cheese Sandwich','Choco biscuit'))

pdf('todos.pdf',width=12,height=6)

par(mfrow=c(1,2))

lineplot.CI(LU..g.fat,Lipolysis,group=FOOD,data=datos2[datos2$Bile.salt.concentration==1 &

datos2$pH.intst==6 & datos2$FOOD %in% c('Greek Yogurt','Pizza','Ham & Cheese Sandwich','Salad'),],

xlab='LU/g fat',ylab='Lipolysis %)',x.leg=2.8,y.leg=30,ylim=c(0,120),las=1)

abline(h=90,lty=2)

lineplot.CI(LU..g.fat,Lipolysis,group=FOOD,data=datos2[datos2$Bile.salt.concentration==1 &

datos2$pH.intst==6 & datos2$FOOD %in% c('Choco biscuit','Milk',

'Bread & butter','Breakfast cereal'),],

xlab='LU/g fat',ylab='Lipolysis (%)',x.leg=2.8,y.leg=30,ylim=c(0,120),las=1)

abline(h=90,lty=2)

dev.off()

o<-lme(Lipolysis~LU..g.fat, random=list(FOOD=pdDiag(~1)),

data=datos2[datos2$Bile.salt.concentration==1 & datos2$pH.intst==6,])

# Read and execute script: "source-segmented.lme.r" (attached at the end)

oseg<-segmented.lme(o, Z=LU..g.fat, random=list(FOOD=pdDiag(~1+G0+U)),psi.link='identity')

oseg

oseg$est.psi

ranef(oseg$lme.fit)

confint(oseg,conf.level = 0.95)

plot(oseg, n.plot=c(2,4), xlim=c(0,4000),ylim=c(0,140),

yscale=-1,leg="top", pch=16, lwd=2,pop=T)

# Source "source-segmented.lme.r"

#############################################################################

## R functions to fit segmented (piecewise linear) mixed models according to:

## Muggeo V., Atkins D.C., Gallop R.J., Dimidjian S. (2014) Segmented mixed models

## with random changepoints: a maximum likelihood approach with application to

## treatment for depression study. Statistical Modelling, 14, 293-313.

## Source this file and read comments and some worked examples at:

## https://www.researchgate.net/publication/292629179

## Please send bugs or comments to: vito.muggeo@unipa.it

## Please cite "Muggeo et al. (2014)" (see above) if you use these functions for your job

#############################################################################

logLik.segmented.lme<-function(object){

a<-logLik(object$lme.fit.noG)

attr(a, "df") <-attr(logLik(object$lme.fit), "df")

a

}

plot.segmented.lme<-function(obj, id=NULL, res=TRUE, pop=FALSE, yscale=1, n.plot, leg="topright", vline=FALSE, ...){

#col modifica solo i punti (ammesso che res=TRUE); le linee sono sempre NERE!!!!!!!

#plotting fitted segmented relationships for multiple subjects

#obj: a "segmented.lme" object

#id: the subjects id to be plotted

#n.plot: a vector to be passed to par(mfrow= (should be coherent with length(id)). If missing, it is computed

# depending on length(id)

#yscale=1 => range for all subjects

# x11() #quartz()?

opz<-list(...)

if(is.null(opz$ylab)) opz$ylab<-all.vars(formula(obj[[1]]))[1]

if(is.null(opz$xlab)) opz$xlab<-obj$namesGZ$nameZ

#--

if(is.null(id)) id<-unique(names(obj$Z))

if(missing(n.plot)) n.plot<- if(length(id)<=1) c(1,1) else c(4, ceiling(length(id)/4))

par(mfrow=n.plot)

id.sx <-1+n.plot[2]*(0:(n.plot[1]-1)) #i grafici di sx

id.bot<- (prod(n.plot):1)[1:n.plot[2]] #i grafici di sotto

par(mar=rep(0,4))

par(oma=c(5,5,1,1))

k<-0

for(i in id){

k<-k+1

plotSegLme(obj, id=i, pop=pop, res=res, xLab="", yLab="", main="", xaxt="n",

yaxt="n", leg=leg, yscale=yscale, vline=vline, ...)

#tt<-axTicks(1) las=2

if(k %in% id.bot) axis(1, cex.axis=.7, at=NULL) else axis(1, labels=FALSE)

if(k %in% id.sx) axis(2, labels=TRUE, cex.axis=.7) else axis(2, labels=FALSE)

}

mtext(opz$xlab, 1, line=3, outer=TRUE)

mtext(opz$ylab, 2, line=3, outer=TRUE)

}

plotSegLme<-function(obj, id, add=FALSE, res=TRUE, pop=FALSE,

yscale=-1, main=paste("id =",id), leg=NULL, vline=FALSE, xLab, yLab, ...){

#Simply plots (or adds) the observed data and the segmented fitted lines for subject 'id'

#---

#obj: an object of class "segmented.lme"

#id: the subject 'id'

#add: if FALSE, a new plot is produced with observations and fitted lines superimposed.

#res: if TRUE the observations (partial residuals) are added; otherwise only the fitted lines

#pop: if TRUE the population-level estimate of the segmented relationship is added..

#yscale if <0, the y-scale refers to the values of 'id' only; otherwise the overall range relevant to *all* subjects (useful for comparisons)

#main: the plot title. It can be ""

#leg: if !NULL it can be one of "top", "topright",... and the id subject is put on the plot.

#vline: if TRUE

#...: argomenti da passare al plot, compresi "col.l" e "lwd.l" che servono per le segmented lines individuali

nomi<-rownames(coef(obj[[2]]))

if(!(id %in% nomi)) stop("unit with specified 'id' is not in the model..")

y<-resid(obj[[2]]) + fitted(obj[[2]])

range.ok<-range(y)

y<-y[names(y)==id]

if(yscale<0) range.ok<-range(y)

range.ok[1]<-if(sign(range.ok[1])>0) range.ok[1]*.99 else range.ok[1]*1.01

range.ok[2]<-if(sign(range.ok[2])>0) range.ok[2]*1.01 else range.ok[2]*.99

x<-obj$Z[names(obj$Z)==id]

opz<-list(...)

opz$x<-x

opz$y<-y

if(!is.null(opz$col.l)){

col.l<-opz$col.l

opz$col.l<-NULL

} else {

col.l<-1

}

if(!is.null(opz$lwd.l)){

lwd.l<-opz$lwd.l

opz$lwd.l<-NULL

} else {

lwd.l<- 2

}

if(missing(yLab)) yLab<-"response"

if(missing(xLab)) xLab<-obj$namesGZ$nameZ

opz$ylab<-yLab

opz$xlab<-xLab

if(is.null(opz$cex)) opz$cex<-1.5

if(is.null(opz$pch)) opz$pch<-19

if(is.null(opz$col)) opz$col<-grey(.7)

if(is.null(opz$ylim)) opz$ylim<-range.ok

opz$main<- if(add) " " else main

if(!is.null(leg)) opz$main<-""

if(!res) opz$type<-"n"

#browser()

if(!add) do.call(plot, opz)

if(!is.null(leg)) legend(leg, legend=paste("id =",id), bty="n")

mu<-fitted(obj[[2]])[names(fitted(obj[[2]]))==id] #? fitted.segmented.lme(fit,1)

psi<- obj$est.psi[[paste(id)]]

m<-cbind(x, mu)

m<-m[order(m[,1]), ]

mL<-m[m[,1]<=psi, ,drop=FALSE]

if(nrow(mL)>1){

fL<-splinefun(mL[,1], mL[,2])

f.psi<-fL(psi)

} else {

mR<-m[m[,1]>=psi, ,drop=FALSE]

fR<-splinefun(mR[,1], mR[,2])

f.psi<-fR(psi)

}

lines(c( m[1,1], psi, m[nrow(m),1]), c( m[1,2], f.psi, m[nrow(m),2]), col=col.l, lwd=lwd.l)

if(vline) segments(psi, par()$usr[3], psi, f.psi, lty=3, col=col.l)

points(psi, par()$usr[3]*1, pch=17, col=col.l, cex=1.2)

#codici vecchi..

# #left side

# mL<-m[m[,1]<=psi, ,drop=FALSE]

# fL<-splinefun(mL[,1], mL[,2])

# new.xL<- c(min(mL[,1]), psi)

# #right side

# mR<-m[m[,1]>=psi, ,drop=FALSE]

# fR<-splinefun(mR[,1], mR[,2])

# new.xR<- c(psi, max(mR[,1]))

# lines(new.xL, fL(new.xL), col=1, lwd=2)

# lines(new.xR, fR(new.xR), col=1, lwd=2)

# if(vline) segments(psi, par()$usr[3], psi, fR(psi), lty=3, col=1)

if(pop){

#mu<-fitted(obj[[2]])[names(fitted(obj[[2]]))==id] #e' fitted.segmented.lme(fit,1)

mu<-fitted(obj, level=0)[names(fitted(obj, level=0))==id]

psi<- obj$fixed.psi[[paste(id)]]

m<-cbind(x, mu)

m<-m[order(m[,1]), ]

# mL<-m[m[,1]<=psi, ,drop=FALSE]

# if(nrow(mL)>1){

# fL<-splinefun(mL[,1], mL[,2])

# f.psi<-fL(psi)

# } else {

# mR<-m[m[,1]>=psi, ,drop=FALSE]

# fR<-splinefun(mR[,1], mR[,2])

# f.psi<-fR(psi)

# }

# lines(c( m[1,1], psi, m[nrow(m),1]), c( m[1,2], f.psi, m[nrow(m),2]), col=col.l, lwd=lwd.l)

#left side

m1<-m[m[,1]<=psi, ,drop=FALSE]

#right side

m2<-m[m[,1]>=psi, ,drop=FALSE]

if(nrow(m1)>0){

f1<-splinefun(m1[,1], m1[,2])

estremo<- if(nrow(m2)>0) psi else min(psi, max(m1[,1]))

new.x1<- c(min(m1[,1]), estremo)

}

if(nrow(m2)>0){

f2<-splinefun(m2[,1], m2[,2])

# new.x1<- seq(psi, max(m1[,1]), l=200)

estremo<- if(nrow(m1)>0) psi else max(psi, min(m2[,1]))

new.x2<- c(estremo, max(m2[,1]))

}

if(nrow(m1)>0){

if(nrow(m1)>1) lines(new.x1, f1(new.x1), col=1, lwd=1.5, lty=2) else

lines(new.x1, c(f1(new.x1)[1], f2(new.x2)[1]), col=1, lwd=1.5, lty=2)

}

if(nrow(m2)>0){

if(nrow(m2)>1) lines(new.x2, f2(new.x2), col=1, lwd=1.5, lty=2) else

lines(new.x2, c(f1(new.x1)[2], f2(new.x2)[2]), col=1, lwd=1.5, lty=2)

}

points(psi, par()$usr[3]*1.015, pch=4, col=1)

# segments(psi, par()$usr[3], psi, f1(psi), lty=3, col=1)

}

}

fitted.segmented.lme<-function(fit, level=1){

#fit: an object of class "segmented.lme"

#What about "fitted(oo$lme.fit.noG)" or "fitted(obj,level=1)+fit$Off"?

#fitted(fitG,level=1)+fit$Off e' proprio uguale a fitted(fit.noG, level=1)

#comunque per level=0 (population parameter) l'identit? non vale, ed ? necessario fare i calcoli

# "manualmente"

obj<-fit[[1]]

level<-deparse(level)

switch(level,

"0"={

leftSlope<-fixef(fit[[2]])[fit$namesGZ$nameZ]

b0<-fixef(fit[[2]])["(Intercept)"]

r<-vector("list", length=length(names(fit$est.psi)))

for(id in names(fit$est.psi)){

diffSlope<-fit$fixed.eta.delta[paste(id)]

Psi<- fit$fixed.psi[paste(id)]

x<-fit$Z[names(fit$Z)==id]

mu<-b0+leftSlope*x+diffSlope*pmax(x-Psi,0)

r[[id]]<-mu

}

mu<-unlist(r)

names(mu)<-names(fit$Z)

# mu<-fitted(obj,level=0) + fit$Off

# if("G0"%in%names(ranef(obj))){

# ni<-tapply(obj$groups[,1], obj$groups[,1], length)

# ki<-rep(ranef(obj)[["G0"]],ni)

# mu<-mu + ki*obj$data[["G0"]]

# }

},

"1"={ mu<-fitted(obj,level=1)+fit$Off #e' proprio uguale a fitted(fit[[2]], level=1)

}

) #end_switch

return(mu)

}

bootNP<-function(fit, B=50, seed=NULL, it.max.b=6){

#Non parametric boot for slme4

#fit: un oggetto di classe "segmented.lme"

#-----------------------

update.lme.call<-function (old.call, fixed., ..., evaluate=FALSE) {

call <- old.call

extras <- match.call(expand.dots = FALSE)$...

if (!missing(fixed.)) call$fixed <- update.formula(call$fixed, fixed.)

if (length(extras) > 0) {

existing <- !is.na(match(names(extras), names(call)))

for (a in names(extras)[existing]) call[[a]] <- extras[[a]]

if (any(!existing)) {

call <- c(as.list(call), extras[!existing])

call <- as.call(call)

}

}

if (evaluate) eval(call, parent.frame()) else call

}

#---------

if(is.null(B) || B<=0) stop("'B>0' is requested")

N<-nlevels(fit$lme.fit$groups[[1]]) #n. of subjects

newData<-fit$lme.fit$data

nomeRispo<-all.vars(formula(fit$lme.fit))[1]

#AGGIUSTA la risposta

newData[,nomeRispo]<-newData[,nomeRispo] + fit$Off

o.b<-fit$boot.call

call.b<-update(object=fit, obj=o.b, data=newD, it.max=it.max.b,

start=list(kappa0=startKappa0,kappa=startingKappa), display=FALSE, evaluate=FALSE)

startingKappa<-extract.psi(fit)

startKappa0<- startingKappa[1]

startingKappa<-startingKappa[-1]

nomiKappa<-names(startingKappa)

nomiKappa<-sapply(strsplit(nomiKappa, "G\\."),function(x)x[2])

names(startingKappa) <- nomiKappa

est<-fixef(fit[[1]])

se<-sqrt(diag(vcov(fit[[1]])))

COEF<-SE<-matrix(,B,length(est))

if(!is.null(seed)) set.seed(seed)

for(i in seq(B)){

#build the boot sample

#idx<-sample(N, replace=TRUE)

idx<-sample(1:N, size=N, replace=TRUE)

newD<-do.call("rbind",lapply(idx, function(x)newData[newData$id==x,]))

newD$y.b<- newD$y

fit.b<-try(suppressWarnings(eval(call.b)), silent=TRUE) #envir=newD)

if(is.list(fit.b)){

Tt<-nlme:::summary.lme(fit.b[[1]])$tTable

COEF[i,]<-Tt[,1] #coef

SE[i,]<-Tt[,2] #se

}

}

r<-list(coef=rbind(est,COEF),se=rbind(se,SE))

r

}

vcov.segmented.lme <-function(object, obj.boot, ...){

opz<-list(...)

if(missing(obj.boot)){

if(is.null(opz$B)) {

r<-object$lme.fit$varFix

} else {

obj.boot<-bootNP(object, B=opz$B, seed=opz$seed, it.max.b=6)

}

r<- var(obj.boot$coef[-1,])

} else {

r<- var(obj.boot$coef[-1,])

}

return(r)

}

confint.segmented.lme<-function(object, conf.level=.95, obj.boot, ...){

opz<-list(...)

if(missing(obj.boot)){

if(is.null(opz$B)) {

r<-object$lme.fit$varFix

SE<-sqrt(diag(r))

est<- object$lme.fit$coef$fixed

zalpha<- -qnorm((1-conf.level)/2)

CIN<-rbind(est - zalpha*SE, est + zalpha*SE)

rownames(CIN) <- paste(100*c((1-conf.level)/2, (conf.level + (1-conf.level)/2)),"%", sep="")

} else {

obj.boot<-bootNP(object, B=opz$B, seed=opz$seed, it.max.b=6)

CIN <-ci.boot(obj.boot, conf.level)

}

} else {

CIN <-ci.boot(obj.boot, conf.level)

}

return(CIN)

}

ci.boot<-function(m, conf.level=0.95){ #era ci.boot() #confint.segmented.lme

#computes three boot CI

#m: object returned by bootNP()

est.orig<-m$coef[1,]

se.orig<-m$se[1,]

zalpha<- -qnorm((1-conf.level)/2)

#percentile

CIt<-CIN<-CIperc<-apply(m$coef[-1,], 2, quantile, prob=c((1-conf.level)/2, (conf.level + (1-conf.level)/2)), na.rm=TRUE)

#Normal-based

SE<-apply(m$coef[-1,],2,sd, na.rm=TRUE)

CIN[1,]<-est.orig - zalpha*SE

CIN[2,]<-est.orig + zalpha*SE

#t-boot

Tdistr<-(m$coef[-1,]-matrix(m$coef[1,],ncol=length(est.orig), nrow=nrow(m$coef)-1, byrow=TRUE))/m$se[-1,]

quantT<-apply(Tdistr,2,quantile, prob=c((1-conf.level)/2, (conf.level + (1-conf.level)/2)), na.rm=TRUE)

CIt[1,]<-est.orig- quantT[2,]*se.orig

CIt[2,]<-est.orig- quantT[1,]*se.orig

ris<-list(norm=CIN, perc=CIperc, t=CIt)

ris

}

extract.psi<-function(obj){

#questa funzione restituisce i "kappa", ovvero i coeff di psi..

nomiG<-obj$namesGZ$nomiG

b<-fixef(obj[[1]])[c("G0",nomiG)]

b

}

logL<-function(fit, metodo=1){

#se metodo=1 takes the logLik from the lme fit without the G variables..

#e<- yy-fit0 #level0 residuals

# -(249/2)*log(2*pi)-.5*determinant(V)$modulus-(t(e)%*%solve(V) %*%e) /2

if(metodo==1) return(logLik(fit$lme.fit.noG))

if(class(fit[[1]])=="lme"){

obj<-fit[[1]]

All<-extract.lmeDesign(obj)

#ZZ<-All$Z

XX<-All$X

yy<-All$y

Off<-fit$Off

V<-mgcv::extract.lme.cov(obj, data=obj$data)

} else {

#ZZ<-fit$Z

XX<-fit$X

yy<-fit$y

V<-fit$V

Off<-fit$Off

}

yy<-yy+Off #true response

idG<-match(c("G0",fit$namesGZ$nomiG), colnames(XX))

idG<-idG[!is.na(idG)]

p<-ncol(XX) #including the changepoint parameters

XX<-XX[,-idG]

b<-fixef(obj)[-idG]

#mu<-fitted.segmented.lme(.., level=0)

#mu<-drop(XX%*%b)

#sum(dmvnorm(yy, mu, V, log=TRUE)) #OK

L<-chol(V) #L'L=V

y1<-backsolve(L,yy,transpose=TRUE)

X1<-backsolve(L,XX,transpose=TRUE)

e1<-y1-drop(X1%*%b)

n<-length(y1)

#NB sum(log(diag(L))) ? uguale a determinant(V, logarithm=TRUE)$modulus/2

ll<- if(fit[[1]]$method=="REML") {

(p-n)*log(2*pi)/2-sum(log(diag(L)))-drop(crossprod(e1))/2-as.numeric(determinant(crossprod(X1))$modulus)/2

} else {-n/2*log(2*pi)-sum(log(diag(L)))-drop(crossprod(e1))/2}

ll

}

bootsegMix<-function(fit,B=10, display=FALSE, metodo=1, frac=1, it.max=6, it.max.b=5, seed=NULL, start=NULL){

#metodo: viene passato alla funzione logL. Se 1 la logL che viene calcolata ? quella della componente

# fit$lme.fit.noG, namely the logLik from the lme fit without the G variables..

#bootRestart for slme4

#fit: un oggetto di classe "segmented.lme" (anche proveniente da un altra "bootsegMix" call)

#frac: size of the boot resample..

#start : un vettor con i nomi (se non fornito gli starting values sono presi da fit)

#-----------------------

update.lme.call<-function (old.call, fixed., ..., evaluate=FALSE) {

call <- old.call

extras <- match.call(expand.dots = FALSE)$...

if (!missing(fixed.)) call$fixed <- update.formula(call$fixed, fixed.)

if (length(extras) > 0) {

existing <- !is.na(match(names(extras), names(call)))

for (a in names(extras)[existing]) call[[a]] <- extras[[a]]

if (any(!existing)) {

call <- c(as.list(call), extras[!existing])

call <- as.call(call)

}

}

if (evaluate) eval(call, parent.frame()) else call

}

#---------

N<-nlevels(fit$lme.fit$groups[[1]]) #n. of subjects

newData<-fit$lme.fit$data

nomeRispo<-all.vars(formula(fit$lme.fit))[1]

#AGGIUSTA la risposta

newData[,nomeRispo]<-newData[,nomeRispo] + fit$Off

o.b<-fit$boot.call

#old: start.psi<-extract.psi(fit)

#old: est.psi<-start.psi["G0"]

#old: call.b<-update(object=fit, obj=o.b, data=newD, psi=est.psi, display=FALSE, evaluate=FALSE)

call.b<-update(object=fit, obj=o.b, data=newD, it.max=it.max.b,

start=list(kappa0=startKappa0,kappa=startingKappa), display=FALSE, evaluate=FALSE)

#mycall$data=quote(gh)

o.ok<-update.lme.call(o.b, fixed.=y~.,evaluate=FALSE)

#old: call.ok<-update(object=fit, obj=o.ok, data=newData, psi=est.psi.b, display=FALSE, evaluate=FALSE)

call.ok<-update(object=fit, obj=o.ok, data=newData, it.max=it.max,

start=list(kappa0=startKappa0.b,kappa=startingKappa.b), display=FALSE, evaluate=FALSE)

all.L<-all.psi<-NULL

it<-0

L0<-L.orig<-logL(fit, metodo=metodo)

if(display){

flush.console()

cat("original data:", 0, " logLik =", formatC(as.numeric(L.orig), 3, format = "f")," psi parms:", formatC(extract.psi(fit),4,format="f"),"\n")

}

if(is.null(start)){

startingKappa<-extract.psi(fit)

startKappa0<- startingKappa[1]

startingKappa<-startingKappa[-1]

nomiKappa<-names(startingKappa)

nomiKappa<-sapply(strsplit(nomiKappa, "G\\."),function(x)x[2])

names(startingKappa) <- nomiKappa

} else {

nomiG<-sapply(strsplit(fit$namesGZ$nomiG, "G\\."),function(x)x[2])

if(length(intersect(names(start), c("G0", nomiG)))!=length(start)) stop("'start' should include all the changepoint parameters")

startKappa0<-start["G0"]

startingKappa<-start[-which("G0"%in%names(start))]

nomiKappa<-names(startingKappa)

}

if(!is.null(seed)) set.seed(seed)

for(i in seq(B)){

#build the boot sample

#idx<-sample(N, replace=TRUE)

idx<-sample(1:N, size=trunc(N*frac), replace=TRUE)

newD<-do.call("rbind",lapply(idx, function(x)newData[newData$id==x,]))

newD$y.b<- newD$y

fit.b<-try(suppressWarnings(eval(call.b)), silent=TRUE) #envir=newD)

if(!is.list(fit.b)){

# fit.b<-NULL

while(!is.list(fit.b)){

idx<-sample(1:N, size=trunc(N*frac), replace=TRUE)

newD<-do.call("rbind",lapply(idx, function(x)newData[newData$id==x,]))

newD$y.b<- newD$y

fit.b<-try(suppressWarnings(eval(call.b)), silent=TRUE) #envir=newD)

}

}

if(is.list(fit.b)){

#old: start.psi.b<-extract.psi(fit.b)

#old: est.psi.b<-start.psi.b["G0"]

startingKappa.b<-extract.psi(fit.b)

startKappa0.b<- startingKappa.b[1]

startingKappa.b<-startingKappa.b[-1]

#NB "nomiKappa" dovrebbero essere sempre gli stessi

names(startingKappa.b) <- nomiKappa

fit.ok<-try(suppressWarnings(eval(call.ok)), silent=TRUE) # data=newData)

L1<-if(is.list(fit.ok)) logL(fit.ok, metodo=metodo) else (-Inf)

} else {

stop("the first bootstrap fit is unsuccessful")

}

if(L0<L1) {

fit<-fit.ok

L0<-L1

}

all.psi[length(all.psi)+1]<-est.psi<-extract.psi(fit)["G0"]

all.L[length(all.L)+1]<-L.ok<-max(L0,L1)

it<-it+1

if(display){

flush.console()

ll<-if(it<10) " logLik =" else " logLik ="

cat("boot resample:", it, ll, formatC(L.ok, 3, format = "f")," psi parms:", formatC(extract.psi(fit),4,format="f"),"\n")

}

startingKappa<-extract.psi(fit)

startKappa0<- startingKappa[1]

startingKappa<-startingKappa[-1]

nomiKappa<-names(startingKappa)

nomiKappa<-sapply(strsplit(nomiKappa, "G\\."),function(x)x[2])

names(startingKappa) <- nomiKappa

} #end boot replicates

fit$history.boot.restart<-cbind(b=1:length(all.psi),psi=all.psi, logL=all.L)

#r<-list(seg.lme.fit=fit, history=cbind(b=1:length(all.psi),psi=all.psi, logL=all.L) )

fit

}

print.segmented.lme<-function(x,...){

# cat("*** Segmented Linear mixed-effects model ***\n")

cat("Segmented Linear mixed-effects model \n")

cat(" psi.link =", x$call$psi.link, "\n")

if(!is.null(x$history.boot.restart)) {

n.sol<-length(unique(x$history.boot.restart[,"psi"]))

cat(" boot restart on", nrow(x$history.boot.restart), "samples; ", n.sol, "different solutions found\n")

}

cat("\n")

print(x[[1]])

}

#--------------------------------------------------------------------------------

segmented.lme <- function(obj, Z, psi, z.psi=~1, x.diff=~1,

random=NULL, #una lista quale 'list(id=pdDiag(~1+x+U+G0))'

random.noG=NULL, #una lista senza G0. Se NULL viene aggiornata la formula di random escludendo "G0"

start.pd=NULL, #una matrice come starting value

psi.link=c("identity","logit"), nq=0, adjust=0,

start=NULL, #*named* list list(delta0, delta, kappa) and the 'delta' component, dovrebbe essere anche

#nominata con i nomi delle variabili in x.diff

data,

fixed.parms=NULL, #a *named* vector meaning the coefficients to be mantained fixed during the estimation

tol=0.0001, it.max=10, display=FALSE){

#obj is the lme fit or simply its call

#random: a list with a formula for the cluster variable 'id' and standard linear variables and "U" and "G0" meaning

# random effects for the difference in slope and changepoint parameters. If it.max=0 the breakpoint is not estimated and

# the formula should not include the term "G0".

#random = list(id=pdBlocked(list(pdDiag(~1+x), pdSymm(~U+G0-1))))

#random = list(id=pdBlocked(list(pdSymm(~1+x), pdSymm(~U+G0-1))))

#random=list(id=pdDiag(~1+weeks+U+G0))

#random=list(id=pdSymm(~1+weeks+U+G0))

#

#Problemi: se control?

#control = list(msVerbose = FALSE, niterEM = 100, opt = "optim")

#

#nq: no. obs che consentono di "invalidare" la stima del breakpoints.

# Ovvero se nq=0, gli \hat{\psi}_i sono annullati se \hat{\psi}_i<=min(Z_i) o \hat{\psi}>=max(z_i)

# se nq>0 gli \hat{\psi}_i sono annullati se \hat{\psi}_i<=min(sort(z)[1:nq]) o \hat{\psi}>= max(rev(z)[1:nq]

#adjust valore numerico (0,1,2).

# Se 0 i psi_i vengono stimati "normalmente" e alla convergenza al vettore numerico dei psi viene assegnato un

# vettore di attributi che serve ad etichettare se il breakpoint ? plausibile o meno (secondo il valore di nq)

# Se 1 i psi ottenuti alla fine dell'algoritm vengono aggiustati secondo il valore di nq. Ad es., se nq=1 il breakpoint

# immediatamente prima del max (o dopo il min) vengono forzati al min/max e cos? sono di fatto annullati; naturalmente il

# modello ? ristimato secondo i nuovi psi. Se 2 l'aggiustamento viene fatto durante l'algoritmo..

#---------------------

require(nlme)

#------------------

update.lme.call<-function (old.call, fixed., ..., evaluate=FALSE) {

call <- old.call

extras <- match.call(expand.dots = FALSE)$...

if (!missing(fixed.)) call$fixed <- update.formula(call$fixed, fixed.)

if (length(extras) > 0) {

existing <- !is.na(match(names(extras), names(call)))

for (a in names(extras)[existing]) call[[a]] <- extras[[a]]

if (any(!existing)) {

call <- c(as.list(call), extras[!existing])

call <- as.call(call)

}

}

if (evaluate) eval(call, parent.frame()) else call

}

#---------------------------------------------------------------------------

f.pd<-function(obj){

#dato un modello lme 'obj' restituisce una matrice pdMat che deve essere utilizzata come componente random

# nelle call "call.ok$random<-list(id=pd)"

pdClasse<-class(obj$modelStruct$reStruct[[1]])[1]

if(pdClasse=="pdBlocked"){ #assumiamo solo 2 blocchi..(? un LIMITE, ma ? facile generalizzare..)

start.v<-unlist(lapply(obj$modelStruct$reStruct[[1]], function(z){as.numeric(z)}))

cl1<-class(obj$modelStruct$reStruct[[1]][[1]])[1]

cl2<-class(obj$modelStruct$reStruct[[1]][[2]])[1]

fo1<-attr(obj$modelStruct$reStruct[[1]][[1]],"formula")

fo2<-attr(obj$modelStruct$reStruct[[1]][[2]],"formula")

no1<-attr(obj$modelStruct$reStruct[[1]][[1]],"Dimnames")[[1]]

no2<-attr(obj$modelStruct$reStruct[[1]][[2]],"Dimnames")[[1]]

pd<-pdBlocked(start.v, pdClass = c(cl1,cl2), nam = list(no1, no2), form=list(fo1, fo2))

} else {

fo<-attr(obj$modelStruct$reStruct[[1]],"formula")

pd <- pdMat(as.numeric(obj$modelStruct$reStruct[[1]]), form = fo, pdClass = pdClasse)

}

pd}

#---------------------------------------------------------------------------

###

if(missing(psi) && it.max==0) stop("Please supply 'psi' with 'it.max=0'")

if("G0"%in%all.vars(attr(random$id,"formula")) && it.max==0) stop("'G0' in the random part is meaningless with 'it.max=0'")

if(!(is.call(obj) || class(obj)=="lme")) stop(" 'obj' should be a lme fit or a lme call")

#if(names(obj$modelStruct[[1]])!="id")

if(is.null(random)) {

random=list(id=pdMat(as.numeric(obj$modelStruct$reStruct[[1]]),

form=attr(obj$modelStruct[[1]][[1]],"formula"),

pdClass=class(obj$modelStruct$reStruct[[1]])[1]))

}

psi.link<-match.arg(psi.link)

logit<-function(xx,a,b){log((xx-a)/(b-xx))}

inv.logit<-function(xx,a,b){((a+b*exp(xx))/(1+exp(xx)))}

#Queste funzioni min1() e max1() restituiscono il "quasi" min o max

if(nq>0){

min1<-function(x,na.rm=FALSE){x<-sort(x)[-(1:nq)];min(x,na.rm=na.rm)}

max1<-function(x,na.rm=FALSE){x<-rev(x)[-(1:nq)];max(x,na.rm=na.rm)}

} else {

min1<-min

max1<-max

}

adjust<-max(min(adjust,2),0) #solo 0,1,2 sono consentiti..

#--------------------------

my.call<-if(is.call(obj)) obj else obj$call

#------------------------

# browser()

if(as.character(my.call$random[[1]])!="list") stop("please, use a list to specify the random part in the 'lme' fit") #vedi gamm() per un approccio piu' elegante?

name.group<-names(eval(my.call$random))

name.Z<-deparse(substitute(Z))

if(is.null(my.call$data)) stop("`obj' should include the argument `data'")

allNOMI<-unique(c(name.Z, name.group, all.vars(my.call$fixed), all.vars(my.call$random),

all.vars(z.psi), all.vars(x.diff)))

formTUTTI<-as.formula(paste("~.+", paste(allNOMI,collapse="+")))

formTUTTI<-update.formula(my.call$fixed, as.formula(paste("~.+", paste(allNOMI,collapse="+"))))

anyFixedG<-FALSE

if(!is.null(fixed.parms)){

name.fixed.butG0<-setdiff(names(fixed.parms),"G0") #nomi dei termini fissi escluso G0

anyFixedG<-if(length(name.fixed.butG0)>=1) TRUE else FALSE #ci sono fixed coef nel submodel of psi?

if(anyFixedG){

formTUTTI<-update.formula(formTUTTI, as.formula(paste("~.+", paste(name.fixed.butG0,collapse="+"))))

}

}

if(missing(data)) {

mf<-model.frame(formTUTTI, data=eval(my.call$data), na.action=na.omit)

} else {

mf<-model.frame(formTUTTI, data=data, na.action=na.omit)

}

nomeRispo<-names(mf)[1]

Rispo<-model.response(mf)

#

Z <- mf[[name.Z]]

id <- mf[[name.group]] #obj$groups[,1]

ni<- tapply(id, id, length) #vector of cluster sizes

N<-length(ni)#n. of clusters (subjects)

n<-length(id) #n. of total measurements

id.x.diff<-FALSE

id.z.psi<-FALSE

#M.z.psi <- mf[all.vars(z.psi)] #

#M.x.diff <- mf[all.vars(x.diff)] #

M.z.psi <- model.matrix(z.psi, data = mf)

if("(Intercept)"%in%colnames(M.z.psi)) M.z.psi<-M.z.psi[,-match("(Intercept)", colnames(M.z.psi)),drop=FALSE]

M.x.diff <- model.matrix(x.diff, data = mf)

if("(Intercept)"%in%colnames(M.x.diff)) M.x.diff<-M.x.diff[,-match("(Intercept)", colnames(M.x.diff)),drop=FALSE]

fixed<-"U+G0" #fixed<-"U"

nomiG<-NULL #se non ci sono explicative nel changepoint (se ci sono poi viene sovrascritto)

namesGZ<-list(nameZ=name.Z)

Offs.kappa<-0

if(NCOL(M.z.psi)>0){

id.z.psi <- TRUE

Z.psi <- data.matrix(M.z.psi)

if(anyFixedG){

if(!all(name.fixed.butG0 %in% colnames(M.z.psi))) stop("variable(s) in 'fixed.parms' should be included in 'z.psi'")

Offs.kappa<-Fixed.z.psi<-drop(Z.psi[, name.fixed.butG0, drop=FALSE]%*% fixed.parms[name.fixed.butG0])

Z.psi<-Z.psi[,setdiff(colnames(Z.psi), name.fixed.butG0), drop=FALSE]

}

if(ncol(Z.psi)>0){

nomiG<-paste("G.",colnames(Z.psi),sep="") #paste("G.",colnames(M.z.psi)[-1],sep="")

namesGZ$nomiG<-nomiG

fixed<-paste(fixed,paste(nomiG,collapse="+"),sep="+")

} else {

id.z.psi <- FALSE

}

} else { #se NCOL(M.z.psi)<=0

if(anyFixedG) stop("variable(s) in 'fixed.parms' should be included in 'z.psi' ")

}

if(NCOL(M.x.diff)>0) {

X.diff <- data.matrix(M.x.diff) #eval(obj$call$data)[,deparse(substitute(x.diff))]

id.x.diff <- TRUE

nomiUx<-paste("U.",colnames(M.x.diff),sep="")

namesGZ$nomiUx<-nomiUx

fixed<-paste(fixed,paste(nomiUx,collapse="+"),sep="+")

}

min.Z<-min1(Z)

max.Z<-max1(Z)

mf["U"]<-rep(1,length(id))

#if(!is.null(obj$data)) my.dd<-cbind(obj$data,my.dd)

if(name.group!="id") mf['id']<-mf[name.group] #costruisci un'altra variabile di clustering con il nome id

mf[name.Z]<- Z

est.kappa0<-TRUE

if("G0" %in% names(fixed.parms)) {

est.kappa0<-FALSE

kappa0<-kappa0Fixed<-fixed.parms["G0"]

}

#==================================================================

#==================================================================

if(est.kappa0){

if(!is.null(start$kappa0)) {

psi<-if(psi.link=="logit") inv.logit(start$kappa0,min.Z,max.Z) else start$kappa0

}

if(missing(psi)){

# formulaFix.Poly<-update.formula(my.call$fixed, paste("~.+",name.Z,"+",paste("I(",name.Z,"^2)",sep="")))

# obj2<-update.lme.call(my.call, fixed = formulaFix.Poly, data=mf, evaluate=TRUE)

# psi<- -fixed.effects(obj2)[name.Z]/(2*fixed.effects(obj2)[paste("I(",name.Z,"^2)",sep="")])

psi<-tapply(Z, id, function(.x) sum(range(.x))/2)

# browser()

if((psi <= min(Z)) || psi>=max(Z)) stop("psi estimated by midvalues is outside the range") #the quadratic fit

}

} else { #se e' fissato e quindi non devi stimarlo

psi<- kappa0

}

psi.new <- psi #stime iniziali

if(length(psi)!=1 && length(psi)!=N) stop("length(psi) has to be equal to 1 or n. of clusters")

if(length(psi) == 1) {

psi.new <- rep(psi.new, N) #subj-specific changepoints

}

psi.ex<-rep(psi.new, ni ) #length = N (n. tot obs)

#----------------------------------------

mf$U<- pmax(0, Z-psi.ex)

formulaFix.noG<-update.formula(my.call$fixed, paste("~.+","U"))

if(id.x.diff){

Ux<- as.matrix(mf$U*X.diff)

colnames(Ux)<-nomiUx

mf<-cbind(mf,Ux) #$Ux<- my.dd$U*X.diff

formulaFix.noG<-update.formula(my.call$fixed, paste(".~.+U+",paste(nomiUx,collapse="+"),sep=""))

}

#se vuoi assumere i psi fissi (it.max=0)

if(it.max==0) {

#aggiorna i random effects. Attenzione in tal caso random deve essere "U" ( o "1").

#Se fosse "U+G0" darebbe errore perch? G0 non esiste

#Oppure dovresti modificare la formula di random,

#attr(random[[1]], "formula")<-update.formula(attr(random[[1]], "formula"), ~.-G0)

formulaRand<-formulaRandOrig<-my.call$random

call.ok<-update.lme.call(my.call, fixed = formulaFix.noG, random=random, data=mf, evaluate=FALSE)

o<-eval(call.ok)

return(o)

} #end if(it.max=0)

#---------------------------------------------------------------------------

#should we fit a preliminary model? extract starting values

start.delta0<-start$delta0

if(id.x.diff) start.delta<-start$delta

need.prelim<- (is.null(start.delta0) || (id.x.diff && is.null(start.delta)))

if(need.prelim){

o<-update.lme.call(my.call, fixed=formulaFix.noG, data=mf, evaluate=TRUE)

delta0i<-unlist(coef(o)["U"]) #length= N

if(id.x.diff) delta<-fixed.effects(o)[nomiUx] #length= n.1

} else {

delta0i<-if(length(start.delta0)==N) start.delta0 else rep(start.delta0,N)

if(id.x.diff) delta<-start.delta[nomiUx]

}

start.kappa<-start$kappa

eta.psi<-0

if(id.z.psi) {

if(is.null(start.kappa)) {

kappa<- rep(0, ncol(Z.psi))

names(kappa)<-nomiG

eta.psi<-rep(0,nrow(Z.psi))

} else {

kappa<-start.kappa

names(kappa)<-paste("G.",names(kappa),sep="")

if((length(kappa)!=NCOL(M.z.psi)) || any(is.na(match(names(kappa), nomiG)))) stop("error in the names/length of start.kappa")

eta.psi <- drop(Z.psi%*%kappa)

}

}

#################################

if(anyFixedG) eta.psi<- eta.psi + Offs.kappa

#Offs.kappa<-data.matrix(mf[name.fixed.butG0])%*%fixed.parms[name.fixed.butG0]

#-----------------------------------------------------------

formulaFix<-update.formula(my.call$fixed, paste(".~.+",fixed))

if(!est.kappa0) formulaFix<-update.formula(formulaFix, .~.-G0)

formulaRand<-formulaRandOrig<-my.call$random

minMax<-cbind(tapply(Z,id,min1),tapply(Z,id,max1)) #matrice nx2 dei min-max

#---------------------------------------------------------

call.ok<-update.lme.call(my.call, fixed = formulaFix, random=random, data=mf, evaluate=FALSE,

control = list(msVerbose = FALSE, niterEM = 100, opt = "optim"))

if(!is.null(start.pd)) call.ok$random<-quote(list(id=start.pd))

#--------------------------------------------------------

kappa0i <- if(psi.link=="logit") logit(psi.ex,min.Z,max.Z) else psi.ex #length=n

if(est.kappa0) kappa0<-mean(kappa0i)

ki<-kappa0i-kappa0

etai<- kappa0i + eta.psi

psi.ex<-if(psi.link=="logit") inv.logit(etai,min.Z,max.Z) else etai #length=n

#----------------------------------------------------------

boot.call<-update.lme.call(my.call, y.b~., data=newData, evaluate=FALSE) #salva la call before modifying obj

it <- 1

epsilon <- 9

obj<-o #serve per estrarre la logLik

b.new<-rep(.1,length(all.vars(formulaFix))) #la risposta conteggiata in all.vars(formulaFix) conta per l'intecetta

while(abs(epsilon) > tol){

DD<-if(psi.link=="logit") (max.Z-min.Z)*exp(etai)/((1+exp(etai))^2) else rep(1,n)

V<-ifelse(Z >psi.ex, -1, 0)

VD <- V*DD

mf$U <- pmax(0, Z-psi.ex)

mf$G0<- rep(delta0i,ni)*VD #rowSums(rep(delta0i,ni)*VD)

if(id.x.diff){

Ux<- as.matrix(mf$U*X.diff)

colnames(Ux)<-nomiUx

mf[,which(names(mf)%in%nomiUx)]<-Ux

deltaMatrix<-cbind(rep(delta0i,ni), matrix(delta,nrow=length(V),ncol=length(delta),byrow=TRUE))

deltaVDx<-deltaMatrix*VD*cbind(1,M.x.diff)

mf$G0<-rowSums(deltaVDx)

}

if(id.z.psi){

G<-cbind(mf$G0,mf$G0*M.z.psi)

colnames(G)<-c("G0",nomiG)

mf[,colnames(G)]<-G

}

dev.old <- obj$logLik

#costruisci l'offset e modifica la risposta..

Off<- if(est.kappa0) -kappa0i*mf$G0 else -ki*mf$G0

if(id.z.psi) Off<- Off - drop(as.matrix(mf[nomiG])%*%kappa[nomiG])

mf[nomeRispo]<-Rispo-Off

# estimate the model

########################################

obj<-eval(call.ok)

########################################

b.old<-b.new

b.new<-fixed.effects(obj)

### if(psi.new>max(Z)| psi.new<min(Z)) stop("estimated psi out of range: try another starting value!")

dev.new <- obj$logLik#sum((fitted(obj)-my.dd[,paste(formula(obj))[2]])^2) #

if(display){

flush.console()

if(it == 1) cat(0," ",formatC(dev.old,3,format="f"),"",

"(No breakpoint(s))","\n")

spp <- if(it < 10) "" else NULL

cat(it,spp,"",formatC(dev.new,3,format="f"),formatC(abs(epsilon),3,format="f"),"\n")

}

epsilon <- abs((dev.new-dev.old)/(dev.old+.1))

#epsilon <- max(abs((b.new-b.old)/b.old))

if(it >= it.max) break

if(abs(epsilon) <= tol) break

it <- it+1

#stopping rules not met: update the estimates

##-------------------------------

#delta0i<-if(inflate.res) inflate.2residuals(obj, coeff=TRUE)[,"U"] else unlist(coef(obj)["U"]) #length=N

if(id.x.diff) delta <- fixed.effects(obj)[nomiUx]

delta0i<-unlist(coef(obj)["U"])

if(est.kappa0){

kappa0.old<-kappa0 #length=1

kappa0 <- fixed.effects(obj)["G0"]

#questo controllo ? sbagliato se link.psi="logit"

#if(kappa0<= min(Z) || kappa0>=max(Z)) stop("estimated psi outside the range")

}

kappa0i.old<-kappa0i #length=n

ki<-if("G0"%in%names(ranef(obj))) unlist(ranef(obj)["G0"]) else rep(0,N)

kappa0i <- kappa0+ki #length=N

#kappa0i <-if(inflate.res) inflate.2residuals(obj, coeff=TRUE)[,"G0"] else unlist(coef(obj)["G0"]) #length=N

kappa0i<-rep(kappa0i,ni) #+ kappa0i.old #length=n

ki<-rep(ki,ni)

etai<-kappa0i

if(id.z.psi) {

kappa.old<-kappa #length=1

kappa<-fixed.effects(obj)[nomiG] #esclude G0..

etai<-etai+drop(Z.psi%*%kappa)

}

if(anyFixedG){

etai <- etai+ Offs.kappa

}

psi.old <- psi.ex #length=n.obs

psi.ex<-if(psi.link=="logit") inv.logit(etai,min.Z,max.Z) else etai #length=n

#eventuale aggiustamento dei psi.

# if(adjust==2){

# id.bp<-I(psi.new>minMax[,1]&psi.new<minMax[,2])

# psi.new[!id.bp] <- tapply(Z,id,max)[!id.bp]# minMax[!id.bp,2]

# }

pd<-f.pd(obj)

call.ok$random<-quote(list(id=pd))

# if(it > it.max) break

# if(abs(epsilon) <= tol) break

} #end_while

#---------------------------------------------------------------------------------------

#Adesso devi fare in modo che le linee *veramente si uniscano (no salti), boot restarting e

#valore di logLik ed infine aggiorna obj<-eval(call.ok)

#browser()

fixed.noG<-if(is.null(nomiG)) update.formula(call.ok$fixed, paste(".~.-G0",sep=""))

else update.formula(call.ok$fixed, paste(".~.-G0-",paste(nomiG, collapse="-"),sep=""))

if(is.null(random.noG)){ #se "random.noG" non ? stato specificato in segmented.lme()

random.noG<-random

#Escludi G0 dalla formula random..

# -

if(!is.null(attr(random[[1]], "formula"))){ #se random e' un'unica formula tipo 'list(id=pdDiag(~1+weeks+U+G0))' (o forse anche 'list(id=pdSymm(~1+weeks+U+G0))'

attr(random.noG[[1]], "formula")<- update.formula(attr(random[[1]], "formula"), ~.-G0)#~1 + weeks + U

} else { #SOLO LA FORMULA DOVE C'E' G0 viene aggiornata, se random ? una lista di formule 'list(id=pdBlocked(list(pdSymm(~1+weeks), pdSymm(~U-1))))'

idRand.G0<-which(sapply(sapply(random[[1]], function(.x) attr(.x,"formula")), function(.x) "G0"%in%all.vars(.x)))

attr(random.noG[[1]][[2]], "formula")<-update.formula(attr(random[[1]][[idRand.G0]], "formula"),~.-G0)

}

# -qui

#attr(random.noG[[1]][[2]], "formula")<-update.formula(attr(random.noG[[1]][[2]], "formula"),~.-G0)

}

call.ok.noG<-update.lme.call(call.ok, fixed = fixed.noG, random = random.noG)

mf[nomeRispo]<-Rispo

obj.noG<-eval(call.ok.noG)

if(it > it.max) warning("max iterations achieved", call. = FALSE)

psi.new<-psi.ex[cumsum(ni)]

names(psi.new)<-levels(unlist(obj$groups))

id.bp<-I(psi.new>minMax[,1]&psi.new<minMax[,2])

#mf$rispo<-Rispo

#o.new<-lme.formula(rispo ~ x + U + U.x.diff, data = mf, random=list(id=pdDiag(~1+x+U)), method=..)

#return(o.new)

if(adjust==1){

#ristima il modello con i nuovi psi ( e le nuove variabili)

psi.new[!id.bp] <- tapply(Z,id,max)[!id.bp]# minMax[!id.bp,2]

psi.ex <- rep(psi.new, aa) #length=n.obs

DD<-fn1(c(rep(kappa0,aa),kappa1), Z.psi ,2, link=psi.link) #length=n.obs

V<-ifelse(Z >psi.ex, -1, 0)

my.dd$U<- pmax(0, Z -psi.ex)

VD <- V*DD

deltaMatrix<-cbind(rep(betaa,aa), matrix(delta,nrow=length(V),ncol=length(delta),byrow=TRUE))

deltaVDx<-deltaMatrix*VD*M.x.diff

G0<-rowSums(deltaVDx)

G<-G0*M.z.psi

colnames(G)<-c("G0",paste("G.",colnames(M.z.psi)[-1],collapse="+",sep=""))

my.dd<-cbind(my.dd, G)

dev.old <- obj$logLik

#stima il modello:

obj<-eval(call.ok)

}

attr(psi.new,which="is.break")<-id.bp

#if(id.z.psi) names(kappa)<- colnames(M.z.psi) #? gi? fatto prima

RIS <- list("lme.fit"=obj, "lme.fit.noG"=obj.noG, "est.psi"=psi.new, call=match.call())

if(!is.null(fixed.parms)) RIS$fixed.parms<-fixed.parms

if(id.z.psi) {

RIS$fixed.eta.psi<-drop(as.matrix(cbind(1,M.z.psi[cumsum(ni),]))%*%c(kappa0,kappa))

names(RIS$fixed.eta.psi) <-names(psi.new)

} else {

RIS$fixed.eta.psi<-rep(kappa0, length(psi.new))

names(RIS$fixed.eta.psi) <-names(psi.new)

}

if(id.x.diff) {

RIS$fixed.eta.delta<-drop(as.matrix(cbind(1,M.x.diff[cumsum(ni),]))%*%fixef(obj)[c("U",nomiUx)])

names(RIS$fixed.eta.delta) <-names(psi.new)

} else {

RIS$fixed.eta.delta<- rep(fixef(obj)["U"], length(psi.new))

names(RIS$fixed.eta.delta) <-names(psi.new)

}

RIS$fixed.psi<-if(psi.link=="logit") inv.logit(RIS$fixed.eta.psi,min.Z,max.Z) else RIS$fixed.eta.psi

#browser()

names(RIS$fixed.psi) <- names(psi.new)

RIS$call$psi.link<-psi.link #in questo modo il nome ? "completo"..

RIS$boot.call<-boot.call

RIS$namesGZ<-namesGZ

RIS$Off<-Off

RIS$rangeZ<- tapply(Z, id, range)

names(Z)<-id

RIS$Z<-Z

class(RIS)<-"segmented.lme"

RIS

}
